# Indiana, Illinois and Kentucky Tri-State Medical Society

**Published:** 1878-01

**Authors:** 


### Society Reports.

*Indiana, Illinois and Kentucky Tri-State Medical Society.*

The Third Annual Session of this Society was held in the city of Evansville, Ind., Oct. 16, 17 and 18, 1877.

The Society was called to order at 11 a. m. by Dr. J. W. Compton, Chairman of the Committee of Arrangements, Dr. G. B. Walker, acting as chairman *pro tem.*, Dr. G. W. Burton, Secretary, Dr. J. W. Singleton, Assistant-Secretary, Dr. F. W. Beard, Corresponding Secretary.

After prayer by Rev. Dr. C. B. H. Martin, Hon. J. J. Kleiner, Mayor, followed with a speech of most cordial welcome in behalf of the city. Dr. M. J. Bray followed with an address in behalf of the profession.

He gave the Society a brief medical and local history of the "Crescent City of the North," with his own experience in the earlier days of Evansville, drawing a comparison between ancient and modern medicine.

(When the doctor located in Evansville the principal hotel was a "one-story log hut.")

Adjourned until 2 p. m.

The Society was called to order pursuant to adjournment, by Dr. W. H. Byford, the President. Dr. J. S. Jewell, of Chicago, being called upon to address the Society, gave a brief extemporaneous account of the vaso-motor nervous system. The

Doctor presented several practical considerations of importance in his remarks.

Dr. W. S. Porter, of St. Louis, Mo., then read a paper on "Syphilis of the Air Passages." After a discussion by Drs. T. Keller of Hot Springs, Ark., Hibberd, of Richmond, Bray, Lenthicum, Walker, Jones and Owen, of Evansville, the opinion prevailed that the disease was transmitted to the offspring by the male parent.

Dr. J. W. Compton then read a paper on "State Medicine and Hygiene;" dwelling particularly on epidemic cholera, small-pox, typhoid fever and epidemic dysentery; urging the necessity of a State Board of Health. The Chair appointed Drs. Denning, of Ill.; Letcher, of Ky., and Beard of Ind., a committee on time and place of next meeting.

Adjourned until 7:30 p. m.

The Society met pursuant to adjournment.

Dr. G. B. Walker read a paper entitled, "Observations and Experience in Practice, Particularly with Reference to Obstetrics;" which included a report of 824 cases, giving the per cent. of different presentations, instrumental cases, eclampsia, post-partum hemorrhage, etc. The President compared it to the relation of a soldier's experience after a long and honorable campaign.

The President appointed the following Committee on Nominations: Drs. Gerrish, Indiana; Pritchell, Kentucky; and Fairbrother, Illinois.

Adjourned to 9 a. m. Wednesday.

### Wednesday—Second Day.

Society called to order at 9 a. m., Dr. Byford in the chair.

Dr. J. F. Hibberd, Richmond, Ind., read a paper on "The Instinctive Operations of the Human System, and how they may be Utilized in the Management of Certain Diseases, and more particularly in Functional Intestinal Constipation."

The Doctor defines instinctive operations thus:

"When a child is born, on the instant it breathes and cries. Both these operations are demonstrated instinctive, because they are performed by a living being from unreasoning impulse, unconsciously and involuntarily. The operations of defecation, micturition, and nursing, which follow in early succession, are of the same nature."

Dr. J. W. Singleton, Paducah, then read an essay on "Social Conservatism."

Adjourned to 1 p. m.

The Society met pursuant to adjournment.

Dr. Singleton (by request) read a paper by Dr. J. L. Cook, Kentucky, on "Malarial Coma."

Dr. J. J. A. Ireland, Louisville, Ky., read his report to the Section on Obstetrics and Gynecology, relating to "Some of the duties of the Obstetrician and Gynecologist." The Doctor drew a pen picture of the great works in this department of medicine.

The Committee on Nominations reported the following officers for the ensuing year:

President, J. F. Hibberd, Richmond, Ind.; First Vice President, F. W. F. Gerrish, Seymour, Ind.; Second Vice President, J. W. Singleton, Paducah, Ky.; Third Vice President, H. C. Fairbrother, East St. Louis, Ill.; Secretary, G. W. Burton, Mitchell, Ind.; Treasurer, F. W. Beard, Vincennes, Ind.

Committee on Publication—Drs. Compton, Walker, and Lenthicum, of Evansville.

Standing Committee: Surgery and Anatomy—J. W. Thompson, Kentucky; Practical Medicine—S. H. Charlton, Indiana; Obstetrics and Gynecology—J. Hale, Kentucky; State Medicine—J. H. Letcher, Kentucky.

Special Committee—Influence of Thermal Waters in the treatment of Disease: J. M. Keller, Hot Springs, Ark.

The report was unanimously adopted.

Dr. Dudley S. Reynold, of Louisville, then read a paper on "Cistoid Cicatrix."

Dr. S. E. Munford, Ind., followed with an essay in answer to the question, "Is meddlesome midwifery bad?" (The discussion growing out of the question would lead young practitioners to believe that the profession are not agreed upon all points.)

The President appointed as a Committee on Necrology, Drs. Hibberd, Smith and Pennington, to report upon the loss of the

Society, in the death of Dr. Ezra Read, and Dr. J. B. Armstrong, of Terre Haute, Ind.

Dr. Edwin Walker then read a report on "Syphilis of the Nervous System."

Adjourned to 7:30 p. m.

At night the old Cumberland Presbyterian church was filled to its utmost capacity, to hear the address of the President, Dr. W. H. Byford, on "Some of the Evils Resulting from our Rapid Advance in Civilization." The Doctor's address was well received by the profession, and by the citizens of Evansville. Adjourned to 9 A.M., 18th.

The Society met pursuant to adjournment, the President in the chair.

Dr. J. H. Letcher, of Ky., read a paper on "Intestinal Inflammation in Children." Dr. J. E. Harper, Ind., presented a report on the "Anomalies of Accommodation and Refraction." Dr. L. B. Harvey, Indianapolis, presented a paper on "The use of the Elastic Ligature in the Treatment of Various Forms of Fistula." Dr. J. M. Keller, Hot Springs, Ark., spoke briefly of the success of the Tri-State Society, and its valuable results to the profession. Dr. T. M. Stevens, Indianapolis, read a paper entitled, "Some Thoughts Connected with the Public Health of Indiana." Dr. G. W. Bental presented a short paper entitled, "Facts for the People." Drs. Stevens, Compton, and Burton, were appointed a committee to prepare the papers on State Medicine, and have them published in the journals.

Dr. Byford introduced his successor, Dr. J. F. Hibberd, of Richmond, Ind., who gratefully acknowledged the honor bestowed on him.

On motion of Dr. Letcher, Ky., the thanks of the Society were unanimously tendered Dr. Byford, for the faithful performance of the duties of his office. Adjourned to meet in the city of Springfield, Ills., 2d Wednesday in Nov., 1878.

